# Molecular Cloning and Expression of Osmotin in a Baculovirus-Insect System: Purified Osmotin Mitigates Amyloid-beta Deposition in Neuronal Cells

**DOI:** 10.1038/s41598-017-08396-x

**Published:** 2017-08-15

**Authors:** Noman Bin Abid, Gwangho Yoon, Myeong Ok Kim

**Affiliations:** Division of Life Science and Applied Life Science (BK 21), College of Natural Sciences, Gyeongsang National University, Jinju, 660-701 Republic of Korea

## Abstract

Osmotin is a pathogenesis-related plant protein, have gained focus of research because of its homology with mammalian adiponectin. The therapeutic properties of osmotin have been explored in recent years as it exhibits neuroprotective effects against amyloid beta-, glutamate- and ethanol-induced synaptic dysfunction and neurodegeneration. In the present study, the full-length gene of the tobacco plant osmotin was cloned and expressed in the Sf9 insect cell line using the baculovirus expression system. *In vitro* analysis of purified Osmotin protein showed excellent cell viability, p-AMPK activation and a reduction in amyloid-beta deposition. Immunofluorescent analysis showed significant reduction in amyloid beta deposition in APP over expressing neuronal cells. Osmotin inhibited amyloid beta deposition by influencing expression of APP processing genes including APP, ADAM 10 and BACE 1. Purified Osmotin showed reduction in amyloid beta deposition in different *in vitro* models as well. Osmotin showed similar mechanism when compared with mammalian adiponectin in different *in vitro* models. The present method will be an excellent approach for the efficient and cost-effective production of the functional protein to be utilized for therapeutic purposes. Reduction in amyloid beta deposition by activation of p-AMPK influencing APP processing genes makes osmotin a potent therapeutic candidate for neurodegenerative diseases.

## Introduction

Osmotin, a 26-kD plant protein, belongs to the fifth class of pathogen-related proteins and exhibits antimicrobial activity through innate immunity in plants via a number of biotic and abiotic signals^1^. It is responsible for resistance against salinity and fungal pathogenesis in plants^2^. Osmotin has also been shown to protect against late leaf spot disease in the peanut plant^3^. Osmotin is reported to be involved in cold and drought stresses in addition to salinity stress^4, 5^. Furthermore, osmotin property as a metabolic modulator have also been characterized^6^.

Structural, biochemical and functional analyses have shown that osmotin has a structural and functional homology with the mammalian adiponectin protein^7–10^. Due to these aspects, osmotin has become a focus of therapeutic research for the last several years. Osmotin has shown remarkable neuroprotective as well as neuroregenerative properties. Studies have demonstrated that osmotin showed a neuroprotective effect against ethanol-induced neurodegeneration in early developmental stages^11^. Osmotin treatment reverses glutamate receptor activation by stimulating the JNK/PI3K/Akt intracellular signalling pathway, reversing synaptic deficit and neuronal apoptosis^12^. Osmotin has been reported to result in improvement in Alzheimer’s disease pathological hallmarks such as amyloid beta, tau phosphorylation-induced memory impairment, and neurodegeneration in the mouse hippocampus^13^. Osmotin has been shown to improve Alzheimer’s disease by inhibiting SREBP2 through the AdipoR1/AMPK/SIRT1 pathway^14^. Due to the therapeutic importance of osmotin, its extraction and purification remain a focus of research.

Several procedures have been devised for the molecular cloning of osmotin. Singh *et al*. reported molecular cloning and expression regulation by abscisic acid (ABA)^15^. Takeda *et al*. sequenced the cDNA nucleotide extracted from tobacco cultured cells^16^. Hu, X and A.S. Reddy reported a cloning and expression strategy of Arabidopsis osmotin in bacteria^17^. The osmotin crystal structure was resolved by Min *et al*.^18^. Molecular cloning and functional characteristics of osmotin from *Solanum nigrum* and soybean in *Escherichia coli* have also been reported^19, 20^. Despite several methods reported in the past regarding the molecular cloning of osmotin, there are several limitations to cloning the complete gene. Complete osmotin gene expression of osmotin from the tobacco plant using callus cell culturing requires several months of cell culturing to extract an optimum amount of osmotin.

Amyloid beta plays a crucial role in neurodegenerative diseases as amyloid plaques are the hallmark of Alzheimer’s disease and play an important role in the onset of the disease^21^. In this study, the amyloid precursor protein (APP) plasmid with Swedish and Indiana mutations was transfected into SH-SY5Y neuroblastoma cells. This plasmid mimics the amyloid-beta deposition in neuronal cells of an Alzheimer’s disease brain. Osmotin has been shown to have a good effect on the cell viability of APP-transfected SH-SY5Y cells. Immunoblot and immunofluorescence results show that purified osmotin has shown significant reduction in beta-amyloid deposition in SH-SY5Y neuroblastoma cells. Purified osmotin shows similar functional property when compared with human adiponectin in different cell lines and experimental condition.

This study addresses a comprehensive protocol to clone the complete osmotin gene, express it in a high-throughput manner in the Sf9 cell line, and purify it using Ni-NTA agarose columns. Purified protein have shown excellent cell viability and functional ability to reduce Amyloid beta deposition by p-AMPK activation. Thus osmotin synthesized in present study is a promising candidate as a therapeutic agent against amyloid beta-induced neurodegenerative diseases.

## Results

The present study deals with successful cloning, expression in Sf9 insect cells and purification^22^. Functional properties of purified osmotin is confirmed in different *in vitro* models of amyloid beta-induced neurodegeneration.

### Molecular cloning of the osmotin gene from *Nicotiana tobacum*

The full-length coding sequence of tobacco (*Nicotiana tobacum*) osmotin cDNA was accessed from GenBank Accession No: M29279.1. Primers with an additional EcoRI restriction enzyme site for ligation were designed. The full-length coding sequence of 732 nucleotides was amplified by polymerase chain reaction (Supp. Fig. 1). The full-length coding sequence was amplified because of the therapeutic potential of the completed protein purified from *Nicotiana tobacum* callus cells that exhibited neuroprotective effects. The amplified product was ligated into the pOET 1N_6X His transfer vector^23^. The purified plasmid was transformed into DH5α cells. The amplified transfer vector was sequenced by sequencing PCR to confirm that the ligated transcript was in frame. The electropherograms showed that the complete sequence of osmotin was ligated into the transfer vector (Supplementary data 1). An in-frame sequence with initiation and termination codons is necessary for the translation of a complete and functional osmotin protein.

### Viral seed production and end-point assay

The transfer vector with the osmotin gene along with the baculovirus DNA was transfected into Sf9 cells. After 3 days, the supernatant containing the viral seed was collected^24^. An end-point assay was performed to check the infection efficiency of the viral seed^25^, thus 1 µl, 10 µl and 100 µl of viral seed was added to 33-mm plates seeded with Sf9 cells. Viral infection increased the cell diameter and halted cell division (Supp. Fig. 2a–d). After 48 hours, signs of infection were evident. The infection rate was analysed by counting infected and uninfected cells. The end-point assay shows the infection efficiency of the viral seed to be used for protein expression (Supp. Fig. 2e). An end-point assay is a convenient method to check the infection efficiency of a viral stock at different dilutions^26^.

A viral plaque assay was performed on serial dilutions of 10^−5^, 10^−6^, 10^−7^, and 10^−8^ of the viral seed. Here, 100 µl of the abovementioned dilution was added into 33-mm cell culture plates seeded with Sf9 cells. The viral titre was calculated to be approximately 3.1 pfu/ml for the amplified viral seed^27^.

### Protein expression and purification

**Figure 1:**
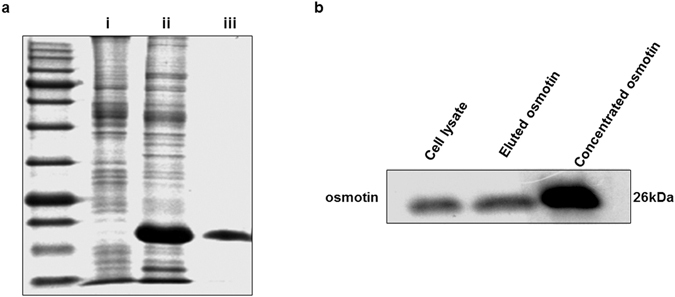
(**a**) Coomassie brilliant blue-stained SDS polyacrylamide gel. (i) Cell lysate of untreated Sf9 cells. (ii) Cell lysate of Sf9 cells expressing osmotin. (iii) Osmotin purified by Ni-NTA agarose columns. (**b**) Immunoblot showing the osmotin protein in the cell lysate and purified osmotin from the Ni-NTA agarose columns.

Sf9 cells cultured in suspension cultured flasks were infected with viral seed at 6 multiplicity of infection (m.o.i.). Cells were cultured for 48 hours, then collected by centrifugation and lysed with cell lysis buffer. The cell lysate was passed through polypropylene columns with (Nickel-nitrilotriacetic acid) Ni-NTA agarose^28^. After appropriate washing to remove debris and impurities, osmotin was eluted from the Ni-NTA agarose by elution buffer with imidazole. Coomassie brilliant blue gels show the expression of the osmotin protein in the infected cell lysate (Fig. 1a). Purified osmotin was further confirmed by Western blot analysis using the osmotin antibody (Fig. 1b).

### Osmotin effected cell viability and proliferation in amyloid beta-induced SH-SY5Y cells

**Figure 2:**
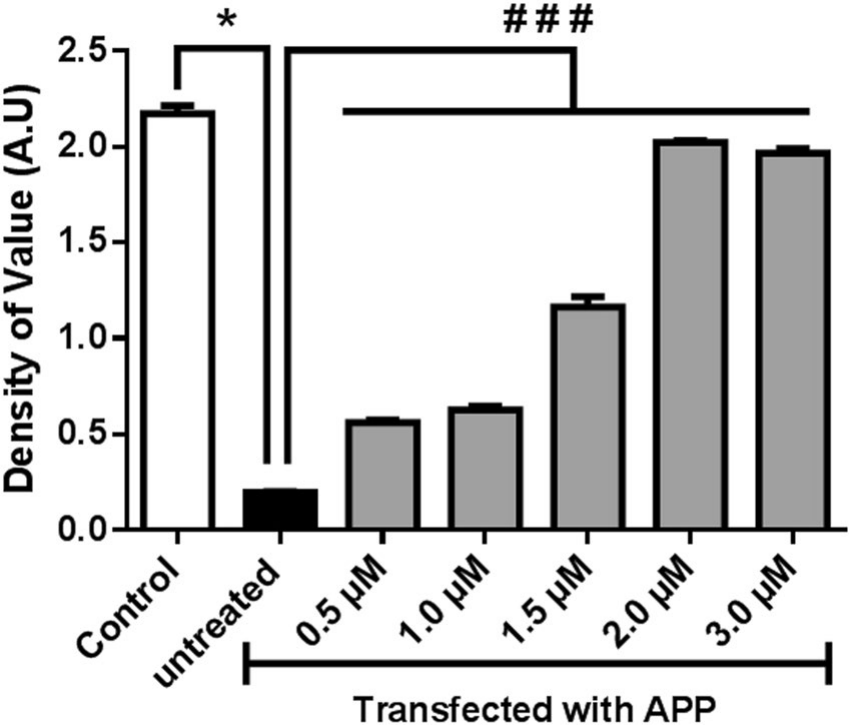
MTT cell proliferation assay showing an increase in the cell proliferation in APP_swe/ind_-overexpressing SH-SY5Y cells. SH-SY5Y cells transfected with the pCAX APP_swe/ind_ plasmid were treated with different concentrations of osmotin: 0.5 µM, 1 µM, 1.5 µM, 2.0 µM, and 3.0 µM. Significant at p < 0.05. *Significantly different from control; ^#^significantly different from the APP-transfected untreated group.

To identify the viable dose of osmotin for neuronal cells, an MTT (3-(4,5-dimethylthiazol-2-yl)-2,5-diphenyltetrazolium bromide) cell proliferation assay was performed^29^. Human neuroblastoma cells seeded in 96-well plates were analysed with different concentrations of osmotin from 0.5 µM to 3.0 µM. The MTT analysis showed that the 2-µM concentration of osmotin had the maximum effect on cell viability and cell proliferation (Fig. 2). MTT is a colourimetric assay that measures the metabolic rate of cells when treated with different concentrations of osmotin. The difference in the metabolic rates of the different groups can be detected by measuring the conversion of purple crystals by the MTT reagent using colourimetric plate readers^30^.

### Osmotin activates p-AMPK in neuronal cells

**Figure 3:**
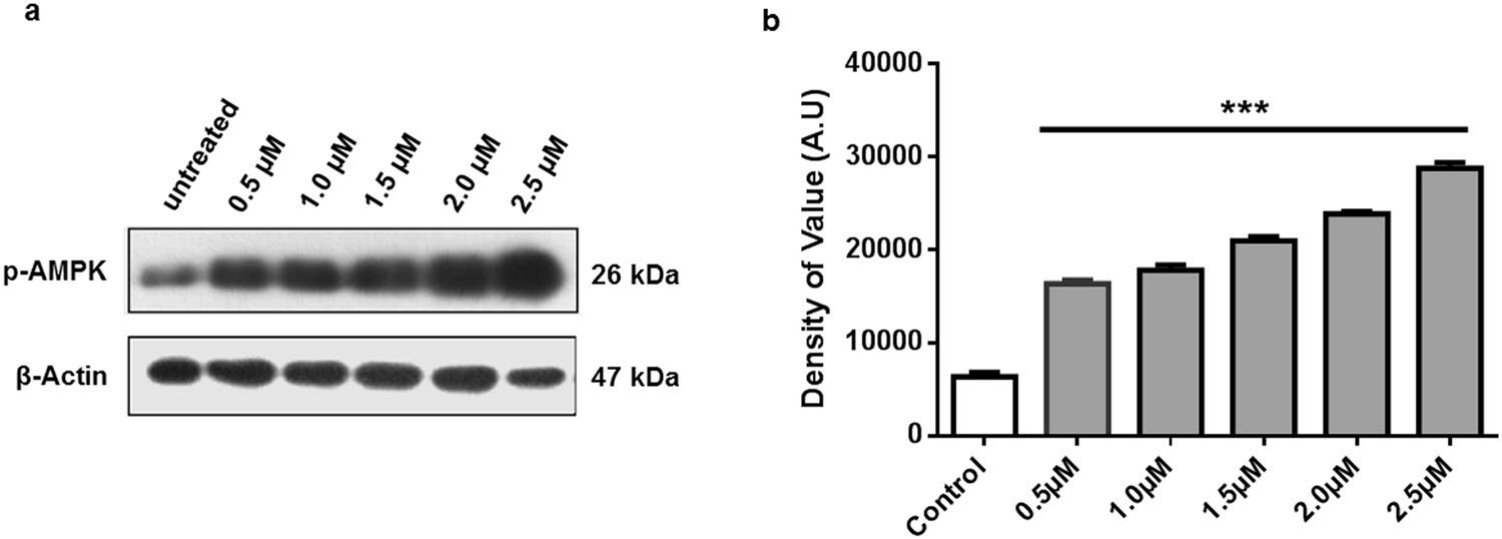
(**a**) p-AMPK activation after treatment with osmotin. *In vitro* treatment with increasing concentrations of osmotin, 0.5 µM, 1 µM, 1.5 µM, 2.0 µM and 2.5 µM, resulted in an increase in p-AMPK activation 20 minutes after treatment. (**b**) Histogram showing the values of p-AMPK activation at different concentrations of osmotin: 0.5 µM, 1 µM, 1.5 µM, 2.0 µM and 2.5 µM. Full-length blots are included in supplementary information file. Significant at p < 0.05. *Significantly different from the control untreated group.

Activation of p-AMPK was analysed by treating SH-SY5Y cells with different concentrations of osmotin to analyse its functional activity. Our results showed an increase in the activation of p-AMPK with increasing concentrations of osmotin from 0.5 µM to 2.5 µM (Fig. 3). Osmotin, as a homologue of adiponectin, should follow the same pathway for the activation of p-AMPK^31^. The whole-cell lysates of cells treated with osmotin were analysed by immunoblot assay using the p-AMPK antibody. The p-AMPK activation assay showed clear evidence of the functional efficiency of purified osmotin.

### Osmotin inhibits amyloid-beta deposition in neuroblastoma cells

**Figure 4:**
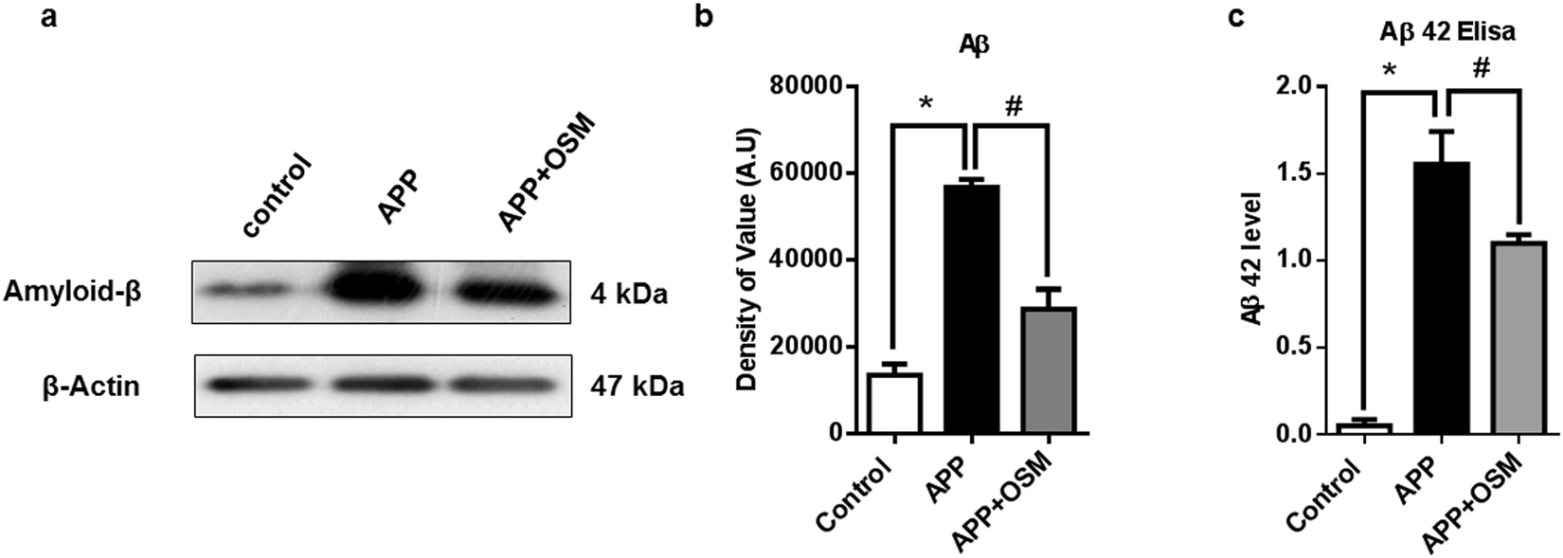
(**a**) Immunoblot showing the effect of osmotin on the amyloid-β concentration in APP_swe/ind_ plasmid-transfected cells. SH-SY5Y cells transfected with the pCAX APP_swe/ind_ plasmid were treated for 24 hours with 2 µM/ml osmotin 72 hours post-transfection. (**b**) Histogram representing the values obtained from the immunoblot assay. (**c**) Histogram representing ELISA results showing Aβ 42 level in pCAX APP_swe/ind_ plasmid-transfected cells. SH-SY5Y cells transfected with the pCAX APP_swe/ind_ plasmid were treated for 24 hours with 2 µM/ml osmotin 72 hours post-transfection. Full-length blots are included in Supplementary information file. Significant at p < 0.05. *Significantly different from the control group. ^#^Significantly different from the APP-transfected group.

Osmotin has been used as a therapeutic agent against amyloid beta-induced neurodegeneration^13^. To further study the efficiency of osmotin expressed in insect cells, SH-SY5Y cells transfected with the APP_swe/ind_ plasmid were treated with osmotin. Transfected cells expressing APP_swe/ind_ mutant gene produces an excessive quantity of amyloid beta thus mimicking the neurodegenerative disease conditions. Cells treated with 2 µM osmotin showed a significant decrease in amyloid beta when analysed with an immunoblot assay (Fig. 4a and b). As beta-amyloid plaques are the hallmark of Alzheimer’s disease pathology^32^, this *in vitro* model mimics the amyloid beta-associated neuropathology. Treatment with osmotin decreased amyloid-beta deposition, further showing its therapeutic activity through influencing amyloid-beta deposition and its associated neuropathy.

The concentration of the amyloid beta (Aβ 42) fragments was confirmed by using a Human Aβ42 ELISA kit. The ELISA results also highlighted the effect of osmotin on amyloid-beta deposition. SH-SY5Y cells transfected with pCAX APP_swe/ind_ were cultured for 72 hours, and the second group of pCAX APP_swe/ind_-transfected cells was treated with osmotin 24 hours prior to cell harvesting. Quantitative analysis of the ELISA showed a significant decrease in Aβ 42 fragments compared to that in the APP_swe/ind_-overexpressing untreated group (Fig. 4c). Thus, the results of quantitative ELISA are in accordance with the immunoblot analysis results.

**Figure 5:**
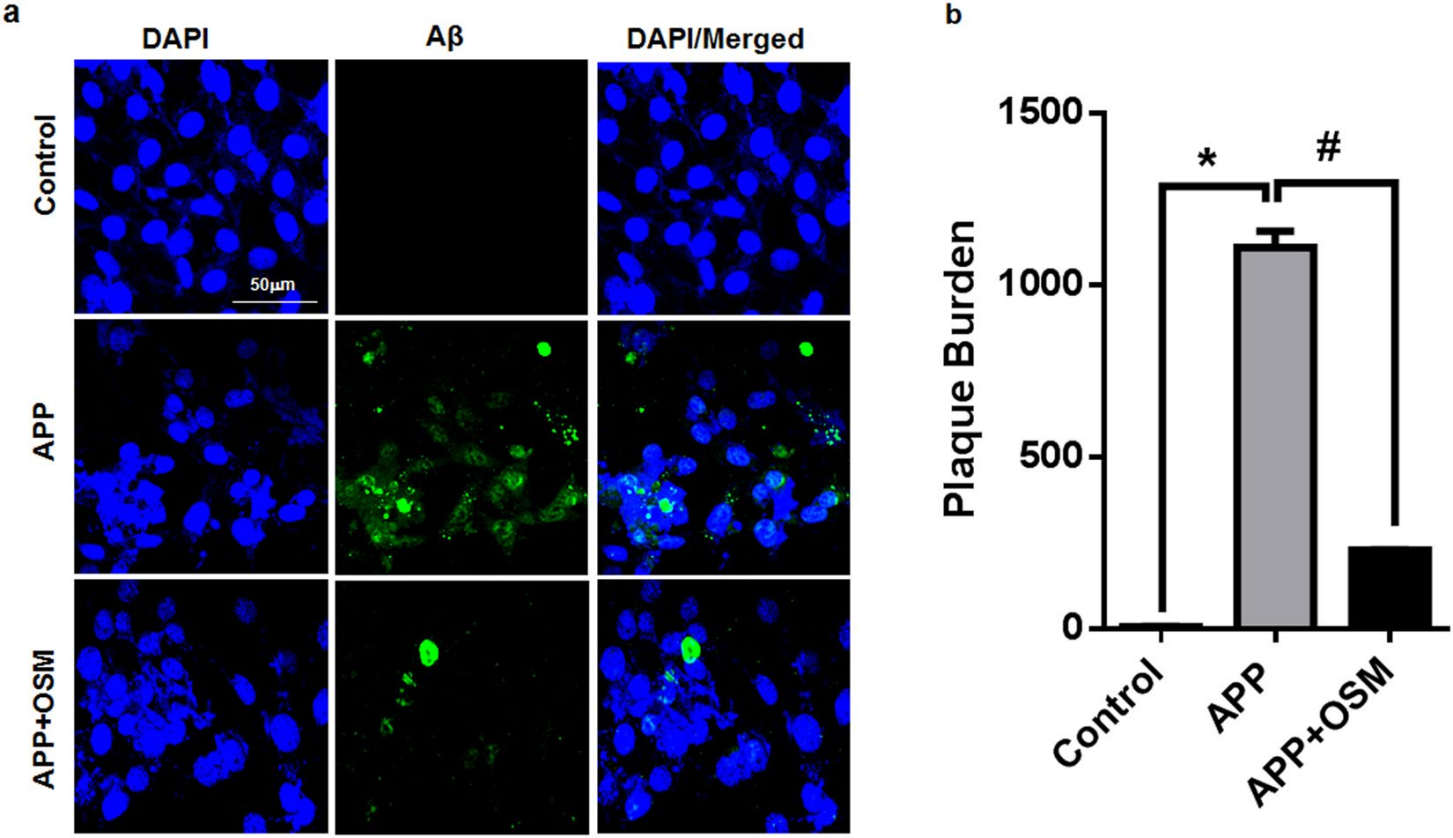
(**a**) Confocal microscope images of the immunofluorescence assay showing Aβ (1–42) conjugated with FITC signals in APP-transfected SH-SY5Y cells. Cells were co-stained with DAPI. (**b**) Histograms showing the plaque percentage in different cells. pCAX APP_swe/ind_ plasmid transfected cells treated with osmotin showed a significant decrease in the plaque intensity compared to that in the untreated APP-transfected cells. Significant at p < 0.05. *Significantly different from the control group. #Significantly different from the APP-transfected group.

The effect of osmotin on the deposition of amyloid beta in the APP-transfected SH-SY5Y cells was confirmed by using an immunofluorescent assay (Fig. 5a). The confocal micrographs indicated that osmotin mitigated the amyloid-beta deposition as the FITC signals in the osmotin-treated group were lower than in the untreated APP-transfected cells. Histograms showed the significant decrease in amyloid beta expression in the osmotin-treated APP-transfected SH-SY5Y cells compared to that in the untreated cells (Fig. 5b).

### Osmotin reduces amyloid-beta deposition by influencing amyloid precursor protein processing proteins

**Figure 6:**
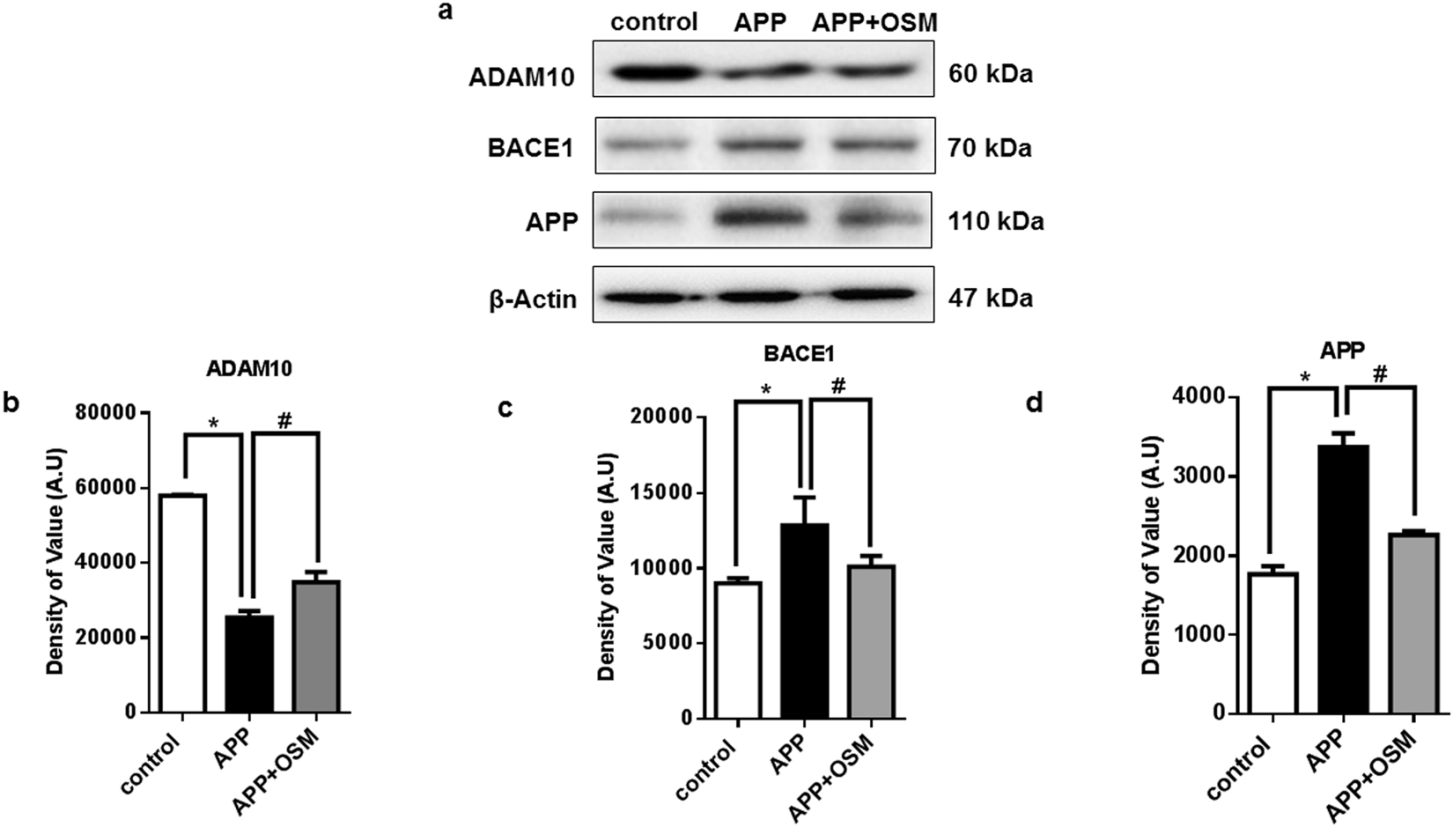
(**a**) Immunoblots showing the protein expression of major APP processing proteins, ADAM 10, BACE1 and APP. The results show the protein expression in the untreated and pCAX APP_swe/ind_ plasmid-transfected cells and the pCAX APP_swe/ind_ plasmid transfected cells treated with osmotin for 24 hours. (**b**) Histogram showing the protein expression of ADAM 10. (**c**) Histogram showing the protein expression of BACE1. (**d**) Histogram showing the protein expression of APP. Full-length blots are included in Supplementary information file. Significant at p < 0.05. *Significantly different from the control group. ^#^Significantly different from the APP-transfected group.

To decipher the role of osmotin in mitigating amyloid-beta deposition in neuronal cells, the proteins involved in APP processing were analysed. ADAM 10, BACE1, and APP expression levels were analysed by using an immunoblotting assay^33^ (Fig. 6a). It was evident that the expression of ADAM 10, which is responsible for proper APP processing had declined. While osmotin treatment rescued and increased the expression compared to un treated APP-transfected cells^34^ (Fig. 6b). Beta-site amyloid precursor protein cleaving enzyme 1 (BACE 1) expression was enhanced in neuronal cells that overexpressed APP, showing the disease phenotype. Osmotin-treated cells showed reduced expression, thus indicating that osmotin regulates APP processing (Fig. 6c). APP expression was also analysed and showed that osmotin treatment of APP-overexpressing cells resulted in a decline in the expression of APP with the help of proteins involved in APP processing (Fig. 6d).

### Osmotin exhibits similar effect as adiponectin in different *in vitro* cell models

**Figure 7:**
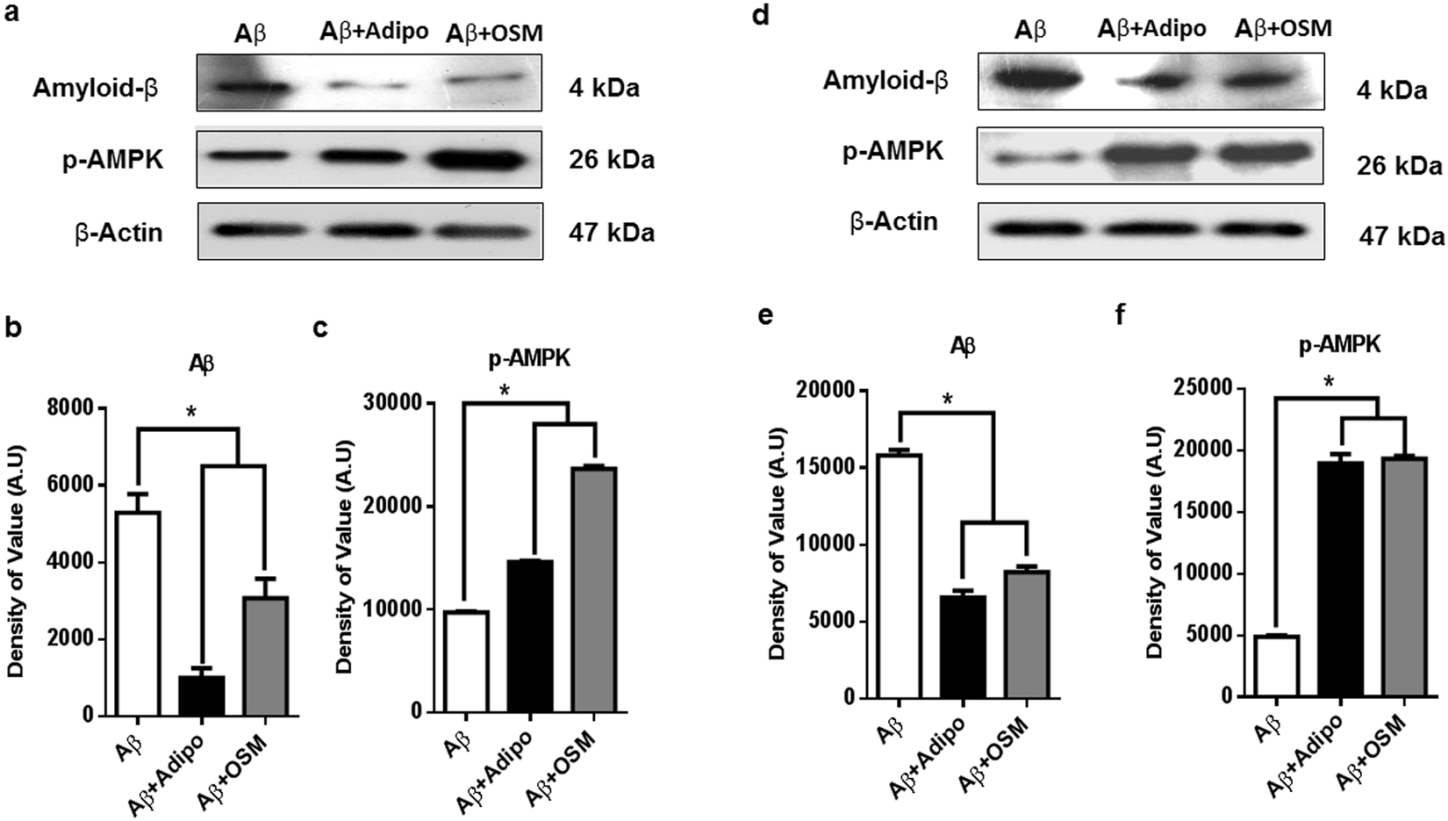
(**a**) Immunoblots showing the protein expression of amyloid beta and p-AMPK protein in HT22 cells exposed to Aβ oligomer and treated with adiponectin and osmotin. (**b**) Histogram showing the protein expression of amyloid beta. **(c)** Histogram showing the protein expression of p-AMPK. (**d**) Immunoblots showing the protein expression of amyloid beta and p-AMPK protein in SH-SY5Y cells exposed to Aβ oligomers and treated with adiponectin and osmotin. (**e**) Histogram showing the protein expression of amyloid beta. (**f**) Histogram showing the protein expression of p-AMPK. Full-length blots are included in a Supplementary information file. Significant at p < 0.05. *Significantly different from the untreated group.

HT22 mouse cells line along with SH-SY5Y cells were cultured in regular growth media. In this experiment, neuronal cells were exposed to 5 μM amyloid beta oligomers. In parallel, cells were co-treated with either 2 µM of osmotin or mammalian adiponectin. Protein expression analysis by immunoblotting showed a decrease in the amyloid beta concentration and an increase in the p-AMPK level in the treatment groups (Fig. 7). Because osmotin is hypothesized to be a homologue of mammalian adiponectin, the effect of both proteins was analysed. The immunoblot showed that there was a significant decrease in the amyloid beta concentration after treatment with osmotin and adiponectin in both HT22 (Fig. 7b) and SH-SY5Y (Fig. 7e) cells. Furthermore, expression of p-AMPK showed a similar pattern of activation in the adiponectin and osmotin treatment groups in HT22 cells (Fig. 7c) and SH-SY5Y cells (Fig. 7f) as well. These results shows that both proteins share similar molecular mechanisms as a similar expression patterns were observed in both HT22 cells and SH-SY5Y cells.

## Discussion

The pharmacological importance of osmotin has been explored because of its structural homology to mammalian adiponectin. Cloning of the complete osmotin gene and its expression in Sf9 insect cells with the help of a baculovirus expression system were performed in this study. The baculovirus expression system is an excellent and convenient system for high-throughput protein expression. Insect cell culturing is convenient and versatile due to its equally efficient growth in both adherent and suspension culturing systems. This system has the capability for large-scale protein production for pharmacological setups. In this study, we used the pOET1N_6x His transfer vector containing a 6xHis tag for separation and purification of the protein by Ni-NTA agarose column purification, which makes the purification step easier and convenient. Overall, the expression and purification process described here is not only easy, scalable, and economical but also quick. These properties make this method more efficient than other methods for the production of osmotin for therapeutic purposes.

Osmotin’s homology with adiponectin makes it a most valuable protein due to its role in metabolic pathways involved in many pathological malfunctions. Adiponectin signalling has been shown not only to play a role in glucose and lipid metabolism but also to have an anti-apoptotic effect by binding adiponectin receptors, which affects S1P, thereby inhibiting apoptosis^35^. Likewise, a role of osmotin in controlling apoptosis through the adiponectin receptor has been observed^7^. Amyloid beta accumulation is the root cause of neurotoxicity and neurodegeneration, which leads to apoptosis, causing memory dysfunction^36, 37^. The present study showed the role of osmotin in rescuing cells from amyloid beta-induced apoptosis. The MTT cell viability assay (Fig. 4) showed an anti-apoptotic effect of osmotin. Cell death was evident in the untreated APP_swe/ind_-transfected SH-SY5Y cells, while an increase in the cell viability was evident with increasing concentrations of osmotin treatment.

Adiponectin plays an important role in metabolic processes through adiponectin receptor-based activation of AMP-activated protein kinase (AMPK). AMPK is known as a master regulator of cellular energy homeostasis^38, 39^. Cellular ATP depletion because of low glucose, hypoxia, or ischemia leads to activation of AMPK^40–42^. AMPK is composed of a catalytic α subunit and regulatory β and γ subunits. Binding of AMP to the γ subunit leads to allosteric activation of the complex. AMPK can also be directly phosphorylated on Thr172 by CAMKK2 in response to changes in intracellular calcium, as occurs following stimulation by metabolic hormones including adiponectin and leptin^43^.

Amyloid precursor protein processing is a major molecular mechanism in neuronal development and functioning^44^. Two of the major players responsible for APP processing, ADAM 10 and BACE1, were analysed in this study. ADAM 10 is involved in normal APP processing and amyloid-beta clearance. In the diseased condition, a decline in ADAM 10 expression causes an amyloid beta burden, which leads to the progression of Alzheimer’s disease^45^. The BACE 1 enzyme is responsible for the cleavage of amyloid precursor protein and is one of the major contributors to APP. Overexpression of APP, which leads to amyloid-beta plaques, is reported to be involved in Alzheimer’s disease^46^. In the present study, ADAM 10 expression was reduced in the APP-overexpression group, while BACE1 expression was enhanced; both of these results showed a diseased phenotype. Osmotin treatment resulted in improvement in the expression of ADAM 10 and lowered the expression of BACE1. APP expression was decrease in the osmotin treatment group, showing that osmotin is responsible for amyloid-beta clearance through APP processing.

Finally, the efficiency of osmotin was analysed in different cell models. Previously, the role for osmotin in the *in vitro* overexpression model was shown in SH-SY5Y cells. To verify the efficiency of osmotin, a different cell line and a different mode of amyloid beta exposure were used. HT22 cells along with SH-SY5Y cells were cultured in normal growth media and then exposed to amyloid beta oligomers. Immunoblot analysis showed that osmotin resulted in an efficient decrease in amyloid beta expression in both cell models, and p-AMPK expression was enhanced in the treatment group.

The functional and therapeutic properties of osmotin were analysed by assessing the cell proliferation and activation of p-AMPK in SH-SY5Y cells after treatment. The increased expression of p-AMPK with an increase in the concentration of osmotin, the decreased amyloid-beta deposition, and enhanced cell survival and viability, as well as the change in the immunofluorescence and expression of APP processing proteins depict the functional aspect of the purified osmotin. The detailed mechanism by which osmotin acts against amyloid beta-inducing neurotoxicity and amyloid-beta clearance is not yet known. Identifying a canonical pathway of osmotin in the treatment of neurodegenerative diseases will be a main component of our future study.

## Materials and Methods

### RNA extraction and cDNA synthesis

RNA was extracted from the *Nicotiana tobacum* plant leaf using an RNeasy Plant Mini Kit (Qiagen, Hilden, Germany). Here, 100 mg of plant leaflets, stem and root tissues were crushed into a fine powder in liquid nitrogen using a mortar and pestle. The osmotin gene from the leaflet was preferred for cloning. cDNA was synthesized using the QuantiTect Reverse Transcription Kit (Qiagen, Hilden, Germany). Genomic DNA was eliminated by adding gDNA wipeout buffer to the mRNA followed by incubation at 42 °C for 2 minutes. Reverse transcriptase enzyme along with RT primers and buffer were added and incubated for 20 minutes at 42 °C, proceeded by deactivation of the reverse transcriptase enzyme by heating at 95 °C for 3 minutes.

### PCR amplification and gene ligation into the transfer vector

Primers were designed for amplification of the osmotin gene. Amplification primers with an additional sequence for the restriction enzyme EcoRI sites were designed to facilitate ligation with the Clonetech In Fusion HD cloning kit (Takara). Forward primer TCGAGAGCTCGAATCCTACTTAGCCAC

TTCATCAC and Reverse Primers TCGAGAGCTCGAATCCTACTTAGCCACTTCATCAC optimised for amplification with the Clone Tech HiFi Polymerase enzyme (Takara) with the following program: 35 cycles of 98 °C for 30 seconds, annealing at 55 °C for 30 seconds and extension at 72 °C for 45 seconds, followed by one cycle of 7 minutes at 72 °C. The purified PCR product and pOET 1N-6xHis transfer vector (Oxford Expression Technology, Oxford, UK) were digested with the EcoRI restriction enzyme followed by ligation with the Clone Tech Infusion HD ligation enzyme (Takara). The ligation product was transformed into DH5α cells. Individual colonies were PCR-amplified for sequencing by pOET sequencing primer (vector (Oxford Expression Technology, Oxford, UK) CAAACTAATATCACAAACTGGAAATGTCTATC. The nucleotide sequence of the PCR product was determined by Macrogen Inc. Korea.

### Cell culturing

The Sf9 insect cell line adapted in serum-free media was cultured in SF-900 II medium (Thermo Fisher Scientific, MA, USA) without serum and antibiotics in a vented-cap suspension cell culture flask at 140–150 rpm at 27 °C without carbon dioxide. SH-SY5Y (ATCC) cells were cultured in an adherent T-75 flask and nourished with Dulbecco’s modified Eagle’s medium (DMEM) (Thermo Fisher Scientific, MA, USA) fortified with 10% foetal bovine serum and 1% penicillin-streptomycin antibiotic. Sf9 cells are easy to culture both in an adherent and suspension culturing condition. The 6x histidine tag was used in the extraction and purification of ultra-pure osmotin with the Ni-NTA purification system^23^.

### Recombinant baculovirus production

To produce recombinant baculovirus, co-transfection was performed in 1 ml serum and antibiotic-free TC100 medium, followed by the addition and mixing of 5 μl transfection reagent FlashFECTIN (Oxford Expression Technologies Oxford, UK) reagent. 100 ng FlashBAC DNA (Oxford Expression Technologies, Oxford, UK) was added along with 500 ng pOET1N-6xHis transfer vector (Oxford Expression Technologies, Oxford, UK). The mixture was then incubated at room temperature for 15–30 minutes after a brief vortex to allow the DNA complexes to form. The transfection mixture was then applied drop by drop to a monolayer of Sf9 cells. Supernatant containing the viral seeds was collected for reinfection and amplification of the viral seed. The viral titre was calculated by viral plaque assay according to a previously explained protocol^24^.

### Purification of 6xHis-tagged proteins from baculovirus-infected insect cells

Ni-NTA agarose is an ideal matrix for the purification of 6xHis-tagged proteins expressed in baculovirus-infected insect cells. Cells collected by centrifugation were lysed by lysis buffer containing 1% Igepal CA-630 (Nonidet P40) and 10 mM imidazole to minimize the binding of the non-tagged, contaminating proteins and to increase the purity with fewer wash steps. The lysate was centrifuged at 10,000 x *g* for 10 minutes at 4 °C to obtain a pellet of cellular debris and DNA. Then, 200 μl 50% Ni-NTA slurry was added per 4 ml of cleared lysate and was gently mixed by shaking (200 rpm on a rotary shaker) at 4 °C for 1–2 h. The Ni-NTA was equilibrated with PBS before it was added to the lysate. The lysate was passed through the column, followed by two washings with wash buffer. The protein was eluted 4 times with 100 μl elution buffer. The protein was concentrated, and imidazole was removed by dialysis using 12-kDa MWCO dialysis tubing.

### APP_swe/ind_ plasmid transfection

pCAX APP_swe/ind_ plasmid, a gift from Dennis Selkoe & Tracy Young-Pearse (Addgene plasmid # 30145), was transfected in 75% confluent SH-SY5Y cells in a 6-well cell culturing plate. Transfection was conducted using Lipofectamine 3000 transfection reagent (ThermoFisher, Waltham, MA, USA) according to the manufacturer’s protocol.

### Western blot analysis

The protein concentration was quantified by using the Bradford assay (Bio-Rad protein assay kit, Bio-Rad Laboratories, CA, USA). Equal amounts of protein (20 µg) were electrophoresed under the same experimental conditions using 10–12% SDS gels and MES SDS running buffer 1x (Novex, Life Technologies, Kiryat Shmona, Israel) with a broad-range prestained protein marker (GangNam stain, Intron Biotechnology, South Korea) as a molecular size control. Membranes were blocked in 5% (w/v) skim milk to reduce non-specific binding and incubated with primary antibodies overnight at 4 °C at 1:1,000-1:10000 dilutions. After the reaction with a horseradish peroxidase-conjugated secondary antibody, as appropriate, the proteins were detected using an ECL detection reagent according to the manufacturer’s instructions (Amersham Pharmacia Biotech, Uppsala, Sweden). The X-ray films were scanned, and the optical densities of the bands were analysed via densitometry using the computer-based ImageJ program.

### Immunofluorescence

The slides were washed twice for 15 minutes in 0.01 M PBS, followed by blocking for 1 hour in 5% normal goat serum. After blocking, the slides were incubated overnight in mouse anti-Aβ (B-4) antibody (Covance, 5858 Horton Street, Suite 500, CA, USA) diluted 1:100 in blocking solution. Following the incubation in the primary antibodies, the sections were incubated for 1.5 hours in FITC goat anti-mouse (1:50) (Santa Cruz Biotechnology, Santa Cruz, CA, USA). After incubation in this secondary antibody, the slides were washed with PBS and mounted with 4,6′-diamidino-2-phenylindole (DAPI) and Prolong Antifade Reagent (Molecular Probe, Eugene, OR, USA). The Aβ staining patterns were examined using a confocal laser-scanning microscope (Flouview FV 1000, Olympus, Japan).

### Enzyme-linked immunosorbent assay

ELISA was conducted by using the Human Aβ42 ELISA kit (Life Technologies, CA, USA) according to the manufacturer’s instructions. The protein sample was treated with serine protease inhibitor to avoid Aβ degradation. Aβ42 concentration was measured by measuring the absorbance at 450 nm through GLOMAX™ Multi detection system (Promega, MDN, USA) plate reader.

### Proteins and Peptides

Aβ _1-42_ peptides and Human Adiponectin recombinant were purchased from Sigma-Aldrich Chemical Co. (St. Louis, MO, USA). Amyloid beta oligomers were prepared according to a previously established protocol^47^.

### Antibodies

The following primary antibodies were used in the Western blot analysis. The osmotin antibody was a kind gift from Dr. Meena from Purdue University, IN, USA; the anti-mouse APP antibody was from Santa Cruz Biotechnology; the anti–p-AMPK and anti-β-actin antibodies were from Cell Signaling Technology, Beverly, MA, USA; the amyloid beta (Aβ) (B-4) (SC-28365, host: mouse monoclonal, 1: 700) antibody was from Santa Cruz Biotechnology.

## Electronic supplementary material

Supplementary information

## References

[1] Viktorova J (2012). Osmotin, a pathogenesis-related protein. Current protein & peptide science.

[2] Subramanyam K (2012). Overexpression of tobacco osmotin (Tbosm) in soybean conferred resistance to salinity stress and fungal infections. Planta.

[3] Vasavirama K, Kirti PB (2012). Increased resistance to late leaf spot disease in transgenic peanut using a combination of PR genes. Functional & integrative genomics.

[4] Patade VY (2013). Cold tolerance in Osmotin transgenic tomato (Solanum lycopersicum L.) is associated with modulation in transcript abundance of stress responsive genes. SpringerPlus.

[5] Goel D, Singh AK, Yadav V, Babbar SB, Bansal KC (2010). Overexpression of osmotin gene confers tolerance to salt and drought stresses in transgenic tomato (Solanum lycopersicum L.). Protoplasma.

[6] Abdin MZ, Kiran U, Alam A (2011). Analysis of osmotin, a PR protein as metabolic modulator in plants. Bioinformation.

[7] Narasimhan ML (2005). Osmotin is a homolog of mammalian adiponectin and controls apoptosis in yeast through a homolog of mammalian adiponectin receptor. Molecular cell.

[8] Arsenescu V (2011). Adiponectin and plant-derived mammalian adiponectin homolog exert a protective effect in murine colitis. Digestive diseases and sciences.

[9] Miele M, Costantini S, Colonna G (2011). Structural and functional similarities between osmotin from Nicotiana tabacum seeds and human adiponectin. PLoS One.

[10] Anil Kumar S, Hima Kumari P, Shravan Kumar G, Mohanalatha C, Kavi Kishor PB (2015). Osmotin: a plant sentinel and a possible agonist of mammalian adiponectin. Frontiers in plant science.

[11] Naseer MI (2014). Neuroprotective effect of osmotin against ethanol-induced apoptotic neurodegeneration in the developing rat brain. Cell death & disease.

[12] Shah SA, Lee HY, Bressan RA, Yun DJ, Kim MO (2014). Novel osmotin attenuates glutamate-induced synaptic dysfunction and neurodegeneration via the JNK/PI3K/Akt pathway in postnatal rat brain. Cell death & disease.

[13] Ali T, Yoon GH, Shah SA, Lee HY, Kim MO (2015). Osmotin attenuates amyloid beta-induced memory impairment, tau phosphorylation and neurodegeneration in the mouse hippocampus. Scientific reports.

[14] Shah, S. A. *et al*. Novel osmotin inhibits SREBP2 via the AdipoR1/AMPK/SIRT1 pathway to improve Alzheimer’s disease neuropathological deficits. *Molecular psychiatry*, doi:10.1038/mp.2016.23 (2016). 10.1038/mp.2016.23 PMC5322276 27001618

[15] Singh NK, Nelson DE, Kuhn D, Hasegawa PM, Bressan RA (1989). Molecular Cloning of Osmotin and Regulation of Its Expression by ABA and Adaptation to Low Water Potential. Plant physiology.

[16] Takeda S, Sato F, Ida K, Yamada Y (1991). Nucleotide sequence of a cDNA for osmotin-like protein from cultured tobacco cells. Plant physiology.

[17] Hu X, Reddy AS (1997). Cloning and expression of a PR5-like protein from Arabidopsis: inhibition of fungal growth by bacterially expressed protein. Plant molecular biology.

[18] Min K (2004). Crystal structure of osmotin, a plant antifungal protein. Proteins.

[19] Campos Mde A (2008). Expression in Escherichia coli, purification, refolding and antifungal activity of an osmotin from Solanum nigrum. Microbial cell factories.

[20] Jami SK, Swathi Anuradha T, Guruprasad L, Kirti PB (2007). Molecular, biochemical and structural characterization of osmotin-like protein from black nightshade (Solanum nigrum). Journal of plant physiology.

[21] Wilkins, H. M. & Swerdlow, R. H. Amyloid precursor protein processing and bioenergetics. *Brain Res Bull*, doi:10.1016/j.brainresbull.2016.08.009 (2016). 10.1016/j.brainresbull.2016.08.009 PMC5316384 27545490

[22] Kovaleva E, Davis DC (2016). Protein Production with Recombinant Baculoviruses in Lepidopteran Larvae. Methods Mol Biol.

[23] Possee RD, King LA (2016). Baculovirus Transfer Vectors. Methods Mol Biol.

[24] King LA, Hitchman R, Possee RD (2016). Recombinant Baculovirus Isolation. Methods Mol Biol.

[25] Bernard, A., Payton, M. & Radford, K. R. Protein expression in the baculovirus system. *Current protocols in neuroscience/editorial board, Jacqueline N. Crawley… [et al.]* Chapter 4, Unit 4, 19, doi:10.1002/0471142301.ns0419s10 (2001). 10.1002/0471142301.ns0419s10 18428480

[26] Hopkins R, Esposito D (2009). A rapid method for titrating baculovirus stocks using the Sf-9 Easy Titer cell line. BioTechniques.

[27] Innami K (2016). Infection studies of nontarget mammalian cell lines with Bombyx mori macula-like virus. Journal of virological methods.

[28] Spriestersbach A, Kubicek J, Schafer F, Block H, Maertens B (2015). Purification of His-Tagged Proteins. Methods in enzymology.

[29] Berridge MV, Herst PM, Tan AS (2005). Tetrazolium dyes as tools in cell biology: new insights into their cellular reduction. Biotechnology annual review.

[30] Berridge MV, Tan AS (1993). Characterization of the cellular reduction of 3-(4,5-dimethylthiazol-2-yl)-2,5-diphenyltetrazolium bromide (MTT): subcellular localization, substrate dependence, and involvement of mitochondrial electron transport in MTT reduction. Archives of biochemistry and biophysics.

[31] Li R (2010). Reduced vascular responsiveness to adiponectin in hyperlipidemic rats–mechanisms and significance. Journal of molecular and cellular cardiology.

[32] Kovacs, G. G. Molecular Pathological Classification of Neurodegenerative Diseases: Turning towards Precision Medicine. *International journal of molecular sciences* **17**, doi:10.3390/ijms17020189 (2016). 10.3390/ijms17020189 PMC4783923 26848654

[33] Chen HY, Wang L, Liu JF, Wang WZ, Yu CJ (2014). Effect of the beta secretase-1 inhibitor on the amyloid C-terminal fragment of amyloid precursor protein processing in a hyperphosphorylated tau rat model. Genet Mol Res.

[34] Harris B, Pereira I, Parkin E (2009). Targeting ADAM10 to lipid rafts in neuroblastoma SH-SY5Y cells impairs amyloidogenic processing of the amyloid precursor protein. Brain Res.

[35] Kadowaki T, Yamauchi T (2011). Adiponectin receptor signaling: a new layer to the current model. Cell metabolism.

[36] Wakx, A. *et al*. Amyloid beta Peptide Induces Apoptosis Through P2X7 Cell Death Receptor in Retinal Cells: Modulation by Marine Omega-3 Fatty Acid DHA and EPA. *Applied biochemistry and biotechnology*, doi:10.1007/s12010-015-1878-6 (2015). 10.1007/s12010-015-1878-6 PMC4718936 26467741

[37] Cieslik M, Czapski GA, Strosznajder JB (2015). The Molecular Mechanism of Amyloid beta42 Peptide Toxicity: The Role of Sphingosine Kinase-1 and Mitochondrial Sirtuins. PLoS One.

[38] Canto C, Auwerx J (2010). AMP-activated protein kinase and its downstream transcriptional pathways. Cellular and molecular life sciences: CMLS.

[39] Mihaylova MM, Shaw RJ (2011). The AMPK signalling pathway coordinates cell growth, autophagy and metabolism. Nature cell biology.

[40] Canto C (2009). AMPK regulates energy expenditure by modulating NAD + metabolism and SIRT1 activity. Nature.

[41] Carling D, Mayer FV, Sanders MJ, Gamblin SJ (2011). AMP-activated protein kinase: nature’s energy sensor. Nature chemical biology.

[42] Hardie DG (2011). AMP-activated protein kinase: an energy sensor that regulates all aspects of cell function. Genes & development.

[43] Kola B, Grossman AB, Korbonits M (2008). The role of AMP-activated protein kinase in obesity. Frontiers of hormone research.

[44] Ramaker JM (2016). Amyloid Precursor Proteins Are Dynamically Trafficked and Processed during Neuronal Development. . Front Mol Neurosci.

[45] Marcello E (2013). Endocytosis of synaptic ADAM10 in neuronal plasticity and Alzheimer’s disease. J Clin Invest.

[46] Munro KM, Nash A, Pigoni M, Lichtenthaler SF, Gunnersen JM (2016). Functions of the Alzheimer’s Disease Protease BACE1 at the Synapse in the Central Nervous System. J Mol Neurosci.

[47] Stine WB, Jungbauer L, Yu C, LaDu MJ (2011). Preparing synthetic Abeta in different aggregation states. Methods Mol Biol.

