# Supplementary material for: Molecular Cloning and Expression of Osmotin in a Baculovirus-Insect System: Purified Osmotin Mitigates Amyloid-beta Deposition in Neuronal Cells
Source: Sci Rep. 2017 Aug 15;7:8147. doi: 10.1038/s41598-017-08396-x (PMC5557928; doi:10.1038/s41598-017-08396-x)

Molecular Cloning and Expression of Osmotin in a Baculovirus-Insect System: Purified Osmotin Mitigates Amyloid-beta Deposition in Neuronal Cells

Noman Bin Abid, Gwang-ho Yoon, and Myeong Ok Kim*

Division of Life Science and Applied Life Science (BK 21), College of Natural Sciences, Gyeongsang National University, Jinju, 660-701, Republic of Korea

* Corresponding author

Myeong Ok Kim, Prof. Ph.D.,

Head of Brain-Metabolic Neurodegenerative Disease Center,

Head of Neuroscience Pioneer Research Center,

Department of Biology and Applied Life Science, College of Natural Sciences,

Gyeongsang National University, Jinju, 660-701, South Korea

Tel.: +82-55-772-1345

Fax: +82-55-772-1349

E-mail: mokim@gnu.ac.kr

Supp. Fig. 1

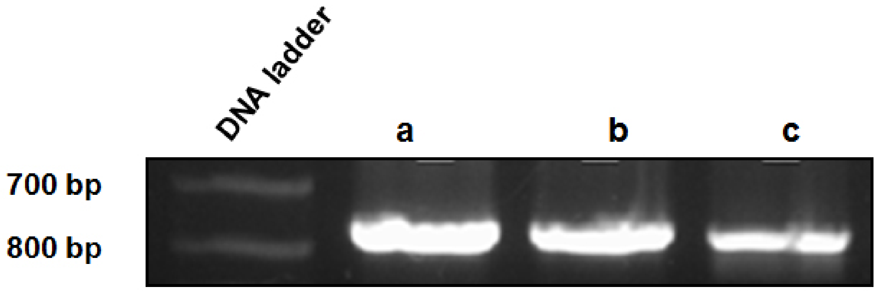

Supplementary Figure. 1: 1.5% electrophoresis agarose gel shows full length PCR amplified product of osmotin gene from leaflets, stem and root respectively. (a-c). Amplified product contain restriction site of EcoR1 to aid ligation into baculovirus transfer vector.

Supp. Fig. 2:

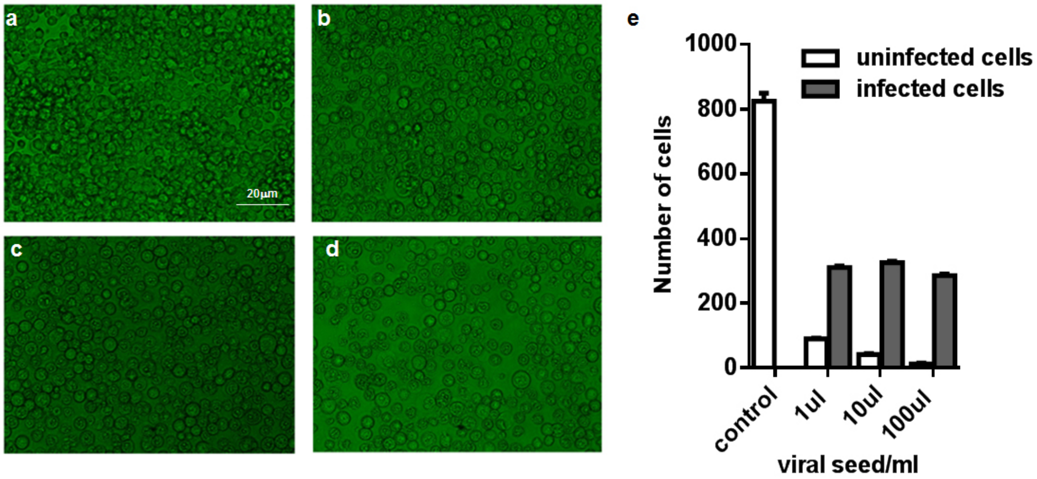

Supplementary figure 2. (a-d) End point assay to detect ability of viral seed to infect sf9 cells at 1µl, 10µl and 100µl per ml. SF9 cells after 72 hours of post infection. Loss of cell division and enlarged nucleus is evident in infected cells. (**e**) Histogram showing comparison of infected and uninfected cells at 1µl, 10µl and 100µl viral seed.

Supplimentary Data 1: Electropherogram showing DNA sequence of Osmotin gene cloned into transfer vector.

File: pOET-OSM_sample_1_poet_Seq_F.ab1 *Signal G:3173 A:5541 C:6236 T:6349* *1716 bases in 21540 scans*

Sample: pOET-OSM_sample_1_poet_Seq_F *Lane: 7* *Base spacing: 14.595694*

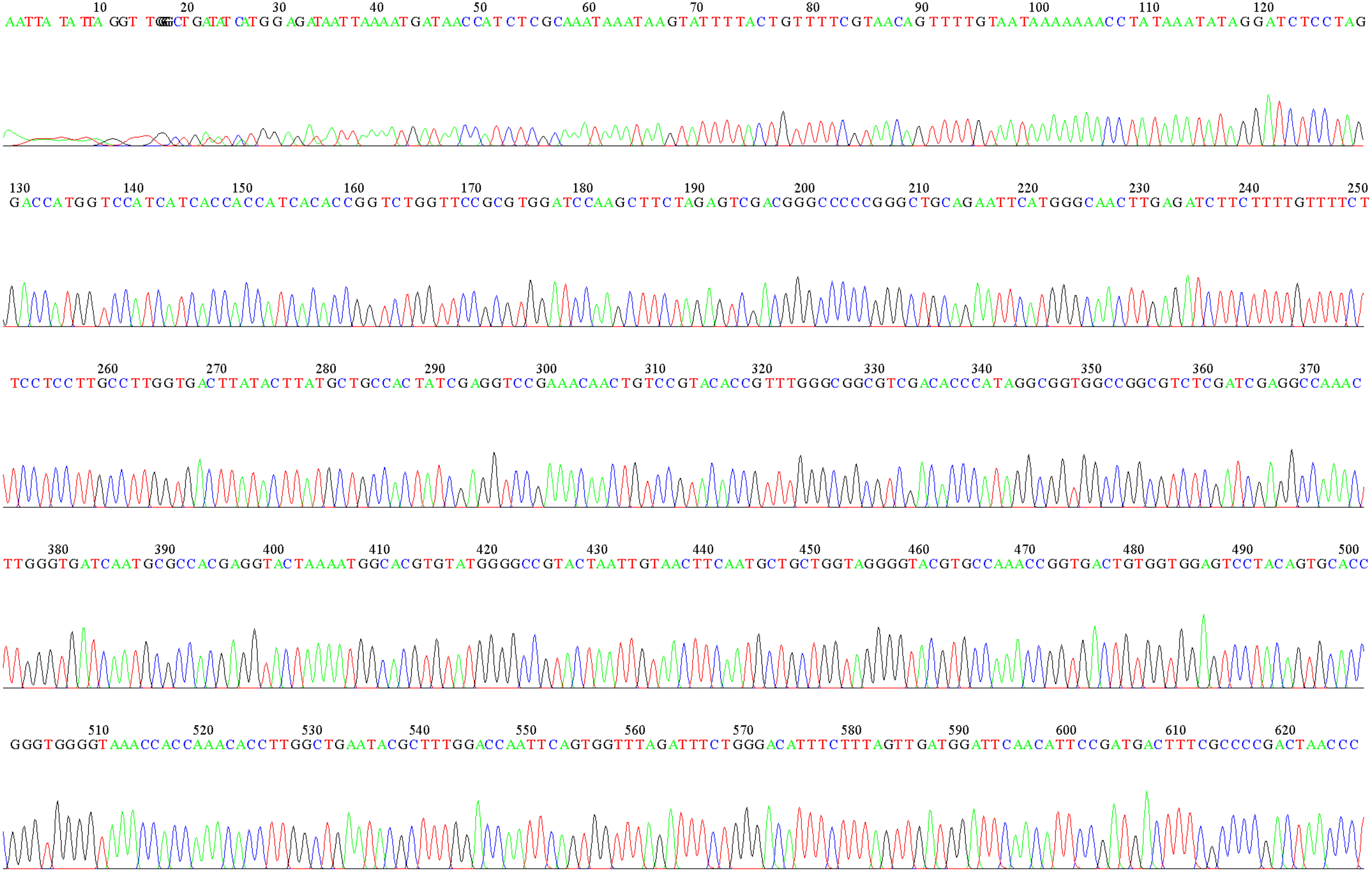

File: pOET-OSM_sample_1_poet_Seq_F.ab1 *Base spacing: 14.595694* *Signal G:3173 A:5541 C:6236 T:6349*

Sample: pOET-OSM_sample_1_poet_Seq_F *Lane: 7* *1716 bases in 21540 scans*

630 640 650 660 670 680 690 700 710 720 730 740 750

TAGTGGAGGGAAATGCCATGCAATTCATTGTACGGCTAATATAAACGGCGAATGTCCCCGCGAACTTAGGGTTCCCGGAGGATGTAATAACCCTTGTACTACATTCGGAGGACAACAATATTGTT

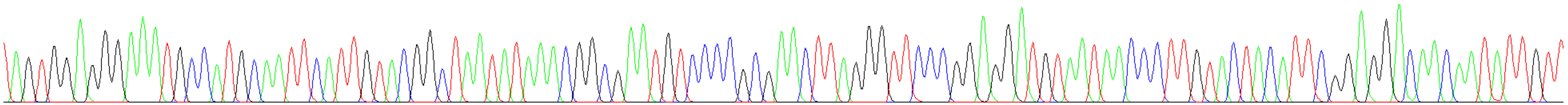

760 770 780 790 800 810 820 830 840 850 860 870

GCACACAAGGACCTTGTGGTCCTACATTTTTCTCAAAATTTTTCAAACAAAGATGCCCTGATGCCTATAGCTACCCACAAGATGATCCTACTAGCACTTTTACTTGCCCTGGTGGTAGTACAA

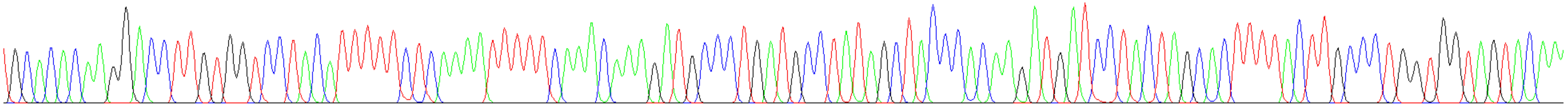

880 890 900 910 920 930 940 950 960 970 980 990

ATTATAGGGTTATCTTTTGTCCTAATGGTCAAGCTCACCCAAATTTTCCCTTGGAAATGCCTGGAAGTGATGAAGTGGCTAAGTAGGAATTCGAGCTCTCGAGGTACCGCGGCCGCAGATCT

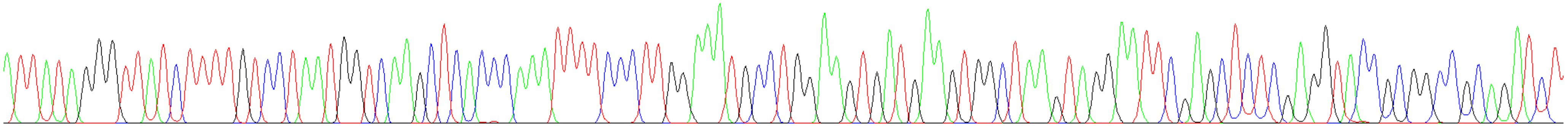

1000 1010 1020 1030 1040 1050 1060 1070 1080 1090 1100 1110

TAATTAATAAAACACGATACATTGTTATTAGTACATTTATTAAGCGCTAGATTCTGTGCGTTGTTGATTTACAGACAATTGTTGTACGTATTTTAAAAATTCATTAAATTATAATCTTTA

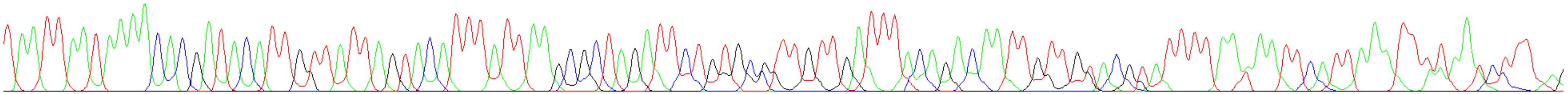

1120 1130 1140 1150 1160 1170 1180 1190 1200 1210 1220 1230

GGGGGGGAAT GTAAAGCGAAAATCAAATGATTTTTCACCGCCTTTTATTCTGAAATTAAAAATTAAAACCTCCAAAAGATTTGTAAAAAAGGTTTCGATTAATTTCTAACCAAGGGTTG

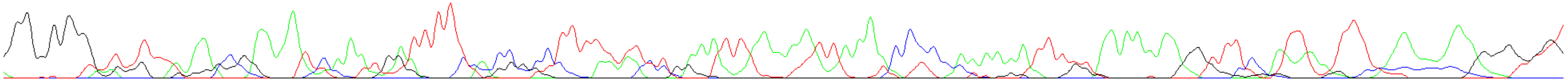

Gels and Multiple Exposure Immunoblots

Figure. 1

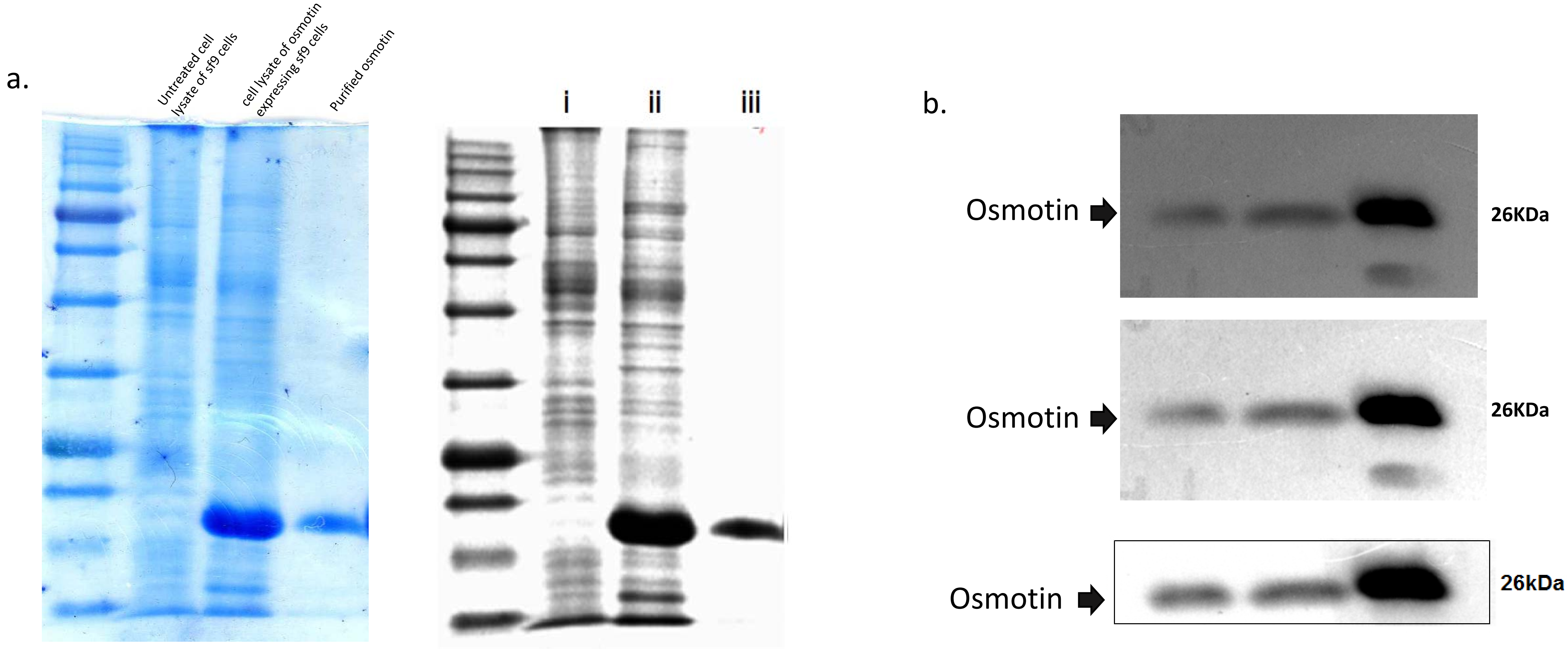

10% SDS gel after Commossie Brilliant Blue staining

Figure. 3

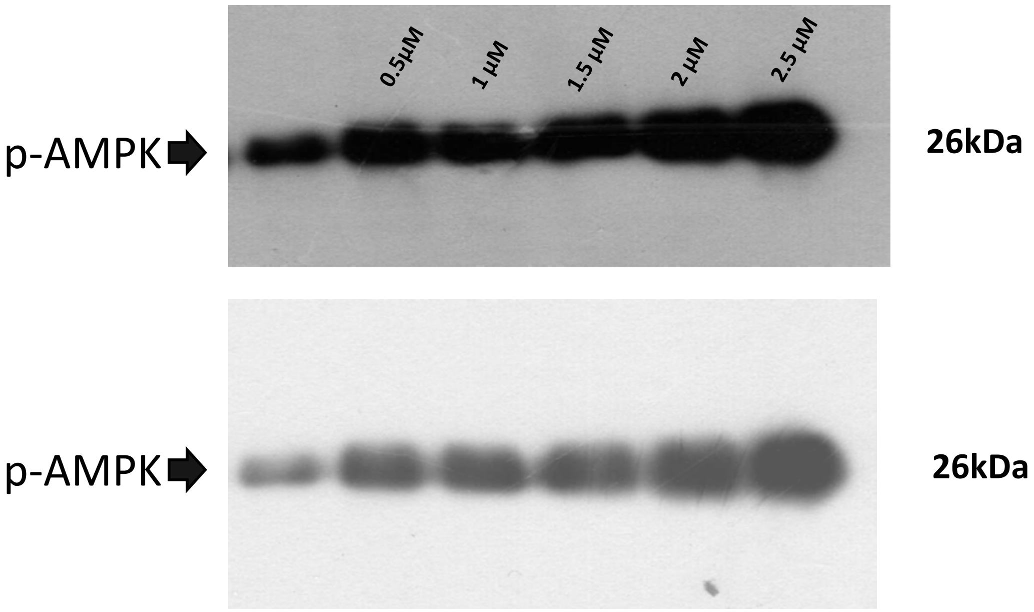

Multiple Exposure immunoblots of p-AMPK

Figure. 4

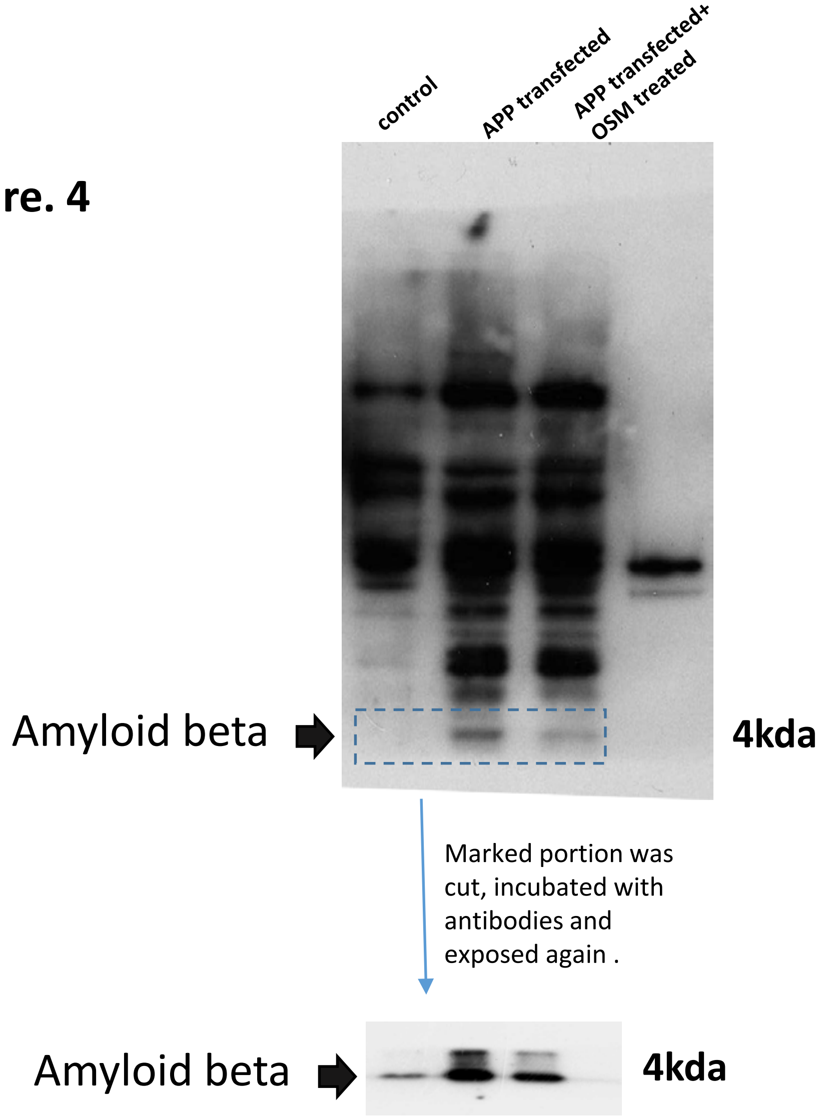

Figure 6

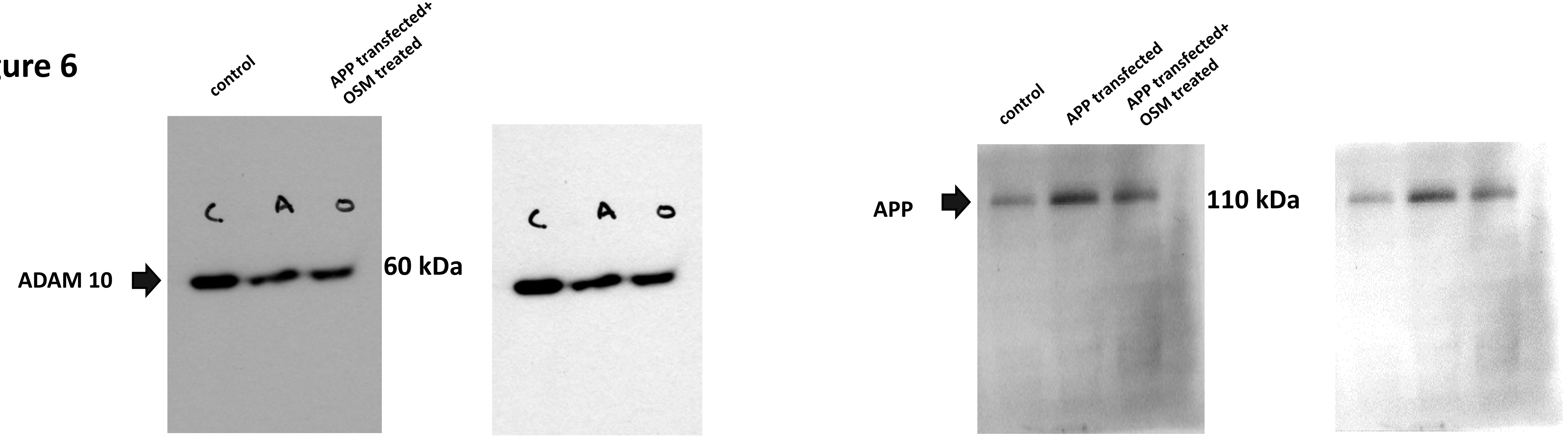

Figure 7

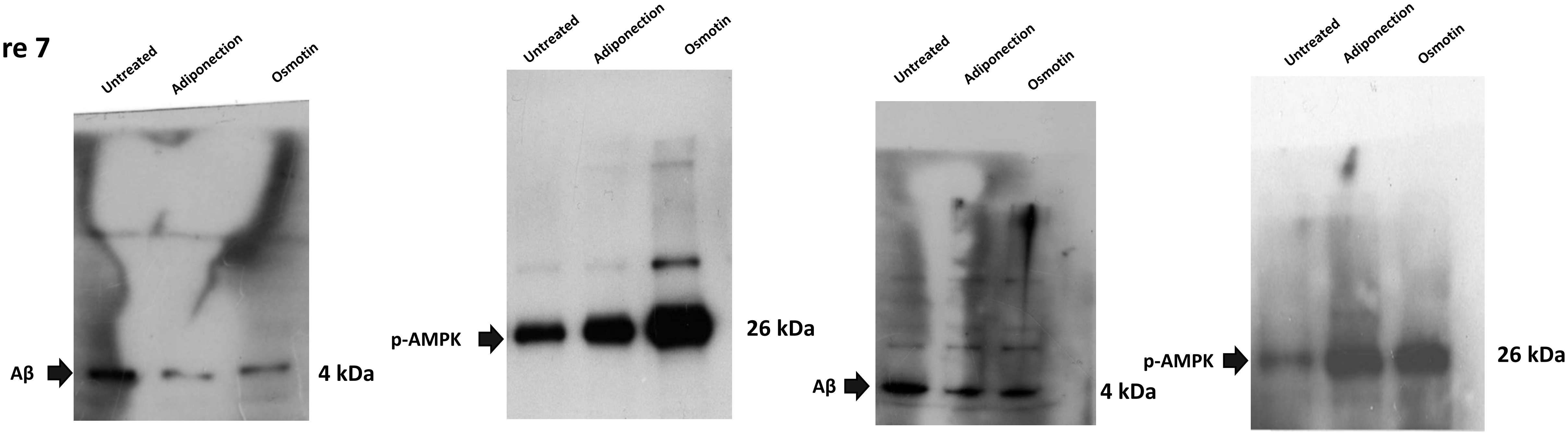

Supplement: Supplementary file 1 — Supplementary information [file 41598_2017_8396_MOESM1_ESM.pdf]
